# Unique Positive Cooperativity Between the *β*-Arrestin–Biased *β*-Blocker Carvedilol and a Small Molecule Positive Allosteric Modulator of the *β*2-Adrenergic Receptor[Fn fn4]

**DOI:** 10.1124/molpharm.121.000363

**Published:** 2021-11

**Authors:** Biswaranjan Pani, Seungkirl Ahn, Paula K. Rambarat, Shashank Vege, Alem W. Kahsai, Andrew Liu, Bruno N. Valan, Dean P. Staus, Tommaso Costa, Robert J. Lefkowitz

**Affiliations:** Department of Medicine (B.P., S.A., S.V., A.W.K., A.L., B.N.V., D.P.S., R.J.L.), Department of Biochemistry (R.J.L.), and Howard Hughes Medical Institute (R.J.L.), Duke University Medical Center, Durham, North Carolina, USA; Department of Medicine, Massachusetts General Hospital, Boston, Massachusetts, USA (P.K.R.); and Viale America 111, Rome, Italy (T.C.)

## Abstract

Among *β*-blockers that are clinically prescribed for heart failure, carvedilol is a first-choice agent with unique pharmacological properties. Carvedilol is distinct from other *β*-blockers in its ability to elicit *β*-arrestin–biased agonism, which has been suggested to underlie its cardioprotective effects. Augmenting the pharmacologic properties of carvedilol thus holds the promise of developing more efficacious and/or biased *β*-blockers. We recently identified compound-6 (cmpd-6), the first small molecule positive allosteric modulator of the *β*2-adrenergic receptor (*β*2AR). Cmpd-6 is positively cooperative with orthosteric agonists at the *β*2AR and enhances agonist-mediated transducer (G-protein and *β*-arrestin) signaling in an unbiased manner. Here, we report that cmpd-6, quite unexpectedly, displays strong positive cooperativity only with carvedilol among a panel of structurally diverse *β*-blockers. Cmpd-6 enhances the binding affinity of carvedilol for the *β*2AR and augments its ability to competitively antagonize agonist-induced cAMP generation. Cmpd-6 potentiates *β*-arrestin1– but not Gs-protein–mediated high-affinity binding of carvedilol at the *β*2AR and *β*-arrestin–mediated cellular functions in response to carvedilol including extracellular signal-regulated kinase phosphorylation, receptor endocytosis, and trafficking into lysosomes. Importantly, an analog of cmpd-6 that selectively retains positive cooperativity with carvedilol acts as a *negative* modulator of agonist-stimulated *β*2AR signaling. These unprecedented cooperative properties of carvedilol and cmpd-6 have implications for fundamental understanding of G-protein–coupled receptor (GPCR) allosteric modulation, as well as for the development of more effective biased beta blockers and other GPCR therapeutics.

**SIGNIFICANCE STATEMENT:**

This study reports on the small molecule–mediated allosteric modulation of the *β*-arrestin–biased *β*-blocker, carvedilol. The small molecule, compound-6 (cmpd-6), displays an exclusive positive cooperativity with carvedilol among other *β*-blockers and enhances the binding affinity of carvedilol for the *β*2-adrenergic receptor. Cooperative effects of cmpd-6 augment the *β*-blockade property of carvedilol while potentiating its *β*-arrestin–mediated signaling functions. These findings have potential implications in advancing G-protein–coupled receptor allostery, developing biased therapeutics and remedying cardiovascular ailments.

## Introduction

G-protein–coupled receptors (GPCRs), also known as seven-transmembrane receptors, constitute the largest family of transmembrane proteins represented in the human proteome. GPCRs are important and ubiquitous portals of cell signaling that are involved in regulating a myriad of physiologic processes (Lefkowitz, 2007; Rockman et al., 2002). The *β*2-adrenergic receptor (*β*2AR) is a widely studied prototypical GPCR, which plays a major role in cardiovascular and pulmonary pathophysiology, along with its closely related *β*1-adrenergic receptor (*β*1AR) subtype. Accordingly, drugs that orthosterically target *β*-adrenergic receptors (*β*-ARs) (*β*-agonists and *β*-blockers) are a current therapeutic mainstay for diseases like asthma and heart failure, respectively (Lohse, 2004; Post et al., 1999; Tilley and Rockman, 2006).

In general, *β*-blockers tend to simultaneously inhibit G-protein and *β*-arrestin signaling downstream of activated receptors. An important exception to this pharmacology is the drug carvedilol, a Food and Drug Administration–approved *β*-blocker used for the treatment of cardiac dysfunctions, including heart failure, hypertension, and postmyocardial infarction (Foody et al., 2002). Unlike other *β*-blockers that are clinically prescribed, carvedilol retains a unique ability to activate *β*-arrestin–mediated signaling while still potently blocking G-protein pathways. Categorically, carvedilol is thus considered to be a GPCR *β*-arrestin–biased ligand, i.e., it can preferentially activate *β*-arrestin but not G-protein–mediated signaling and thereby elicit a phenomenon referred to as “biased agonism” or “functional selectivity” (Smith et al., 2018; Whalen et al., 2011; Wisler et al., 2014). Of note, clinical studies suggest that carvedilol may be superior to other *β*-blockers in preventing heart failure exacerbations and improving overall mortality in patients with reduced heart function (Bristow et al., 1996; Colucci et al., 1996; Packer et al., 1996). Although not firmly established, it has been speculated that these cardioprotective effects of carvedilol may be attributable to its unique ability to activate *β*-arrestin–mediated signaling pathways. The therapeutic implications of biased agonism may be widespread, as biased ligands can refine GPCR functions to regulate only a subset of signaling pathways with desired physiologic outcomes. As such, augmenting the therapeutic profile of carvedilol might lead to the development of improved *β*-blocker therapeutics potentially even with biased properties.

Allosteric modulators could potentially facilitate the improvement of the pharmacological properties of carvedilol. Compared with orthosteric drugs, allosteric ligands bind at receptor sites that are evolutionarily less conserved and topographically distinct from the endogenous ligand binding site and play critical roles in regulating the functional repertoire of orthosteric ligands. Allosteric ligands mediate their cooperative effects through the selection or stabilization of specific conformations in the ensemble of a given GPCR bound to an orthosteric ligand (Thal et al., 2018; Wootten et al., 2013). As modulators of orthosteric ligand function, allosteric ligands are broadly classified as either positive allosteric modulators (PAMs), which potentiate agonist responsiveness of a receptor, or negative allosteric modulators (NAMs), which noncompetitively oppose receptor activation by agonists. In addition to their pharmacological function as PAMs or NAMs, allosteric modulators may also engender biased signaling by virtue of differential cooperative interactions with orthosteric ligands for distinct transducer coupling to the receptor (Gentry et al., 2015; Wootten et al., 2018). We recently isolated several small molecule PAMs and NAMs of the *β*2AR by applying an affinity-based screening strategy on highly diverse DNA-encoded chemical libraries. Of the many small molecule hits obtained from our screens, we identified compound-15 as a lead NAM and compound-6 (cmpd-6) as a lead PAM of the *β*2AR (Ahn et al., 2017; Ahn et al., 2018). Whereas compound-15 allosterically blocks agonist functions at the *β*2AR, cmpd-6 conversely enhances agonist, but not antagonist, functions at the receptor (Liu et al., 2017; Liu et al., 2019). Although allosteric ligands continue to evolve as candidates for the development of next-generation therapeutics, the prospect of allosteric modulation of carvedilol function remains unexplored and is an attractive drug discovery avenue. In this study, we present the discovery of an unexpected unique phenomenon of positive cooperativity between the PAM cmpd-6 and the *β*-arrestin–biased *β*-blocker carvedilol at the *β*2AR. The findings reported herein have important implications for understanding the potential relationships between allostery and biased agonism at GPCRs.

## Materials and Methods

### Reagents

All reagents used in this study are of molecular biology grade. All chemicals and ligands were obtained from Sigma-Aldrich (St. Louis, MO), unless mentioned otherwise. Cmpd-6 (Ahn et al., 2018) and its analogs (A1–A12) were synthesized in-house as reported earlier (Liu et al., 2019). Nanobody-6B9 (Nb6B9) (Ring et al., 2013), heterotrimeric Gs-*αβγ* (Rasmussen et al., 2011b), and a minimal cysteine *β*-arrestin1 (Shukla et al., 2013) truncated at residue 393 were purified following methods described earlier.

### Cell Culture

HEK293 and U2OS cells (American Type Culture Collection, Manassas, VA) were cultured in standard tissue culture incubator maintained at 37°C and 5% CO_2_ under humidified condition. HEK293 and U2OS cells were cultured in minimum Eagle’s medium (MEM) or Dulbecco’s modified Eagle’s medium (DMEM), respectively, supplemented with 10% fetal bovine serum and 1× penicillin/streptomycin mix. HEK293 cell lines stably expressing the GloSensor (Promega, Madison, WI) cAMP reporter or the *β*2AR were maintained as described before. The HEK293 cell line used for total receptor endocytosis was obtained from Eurofins (St. Charles, MO) and maintained according to the manufacturer’s recommendation. Clonal HEK293 cells stably expressing the FLAG-*β*2AR or FLAG-*β*2AR–yellow fluorescent protein (YFP) (Han et al., 2012) and U2OS cells stably expressing *β*2V2R [a chimeric *β*2AR with C-terminal tail of the vasopressin 2 receptor (V2R)] (Oakley et al., 1999) were generated and maintained under G418 selection. Expi293F suspension cells were cultured as per manufacturer’s instructions (Invitrogen, Waltham, MA) in shaker incubator maintained at 37°C and 8% CO_2_ under humidified condition.

### Receptor Purification and High-Density Lipoprotein Reconstitutions

Full-length, N-terminal FLAG-tagged wild-type human *β*2AR was expressed in Sf9 insect cells using recombinant baculovirus and purified by n-dodecyl-*β*-d-maltopyranoside (DDM; Anatrace, Inc., OH) solubilization using anti–FLAG-M1 and alprenolol-ligand affinity chromatography followed by size-exclusion chromatography (SEC) as previously described (Kobilka, 1995). The *β*2AR with the sortase consensus site (LPETGHH) inserted after amino acid 365 was expressed in Expi293F suspension cells (Invitrogen) and purified using anti–FLAG-M1 affinity chromatography. Enzymatic (sortase) ligation of a synthetic phosphopeptide (V_2_Rpp) was done following methods established in previous study (Staus et al., 2018) to generate *β*2AR-pP. Wild-type human *β*1AR, with an N-terminal FLAG-tag, was expressed in Expi293F cells by transient transfections using Expifectamine following manufacturer’s instructions (Invitrogen) and purified using methods established for the *β*2AR (Choi et al., 2018; Staus et al., 2018). In brief, *β*1AR-transfected cells were grown for 60 hours in the presence of alprenolol (2 μM), harvested, and solubilized in lysis buffer containing 1% DDM and 0.05% cholesteryl hemisuccinate. Clarified lysates were passed through anti–FLAG-M1 affinity column, washed, and eluted in cold elution buffer (20 mM HEPES, pH 7.4, 100 mMNaCl, 0.1% DDM, 0.01% cholesteryl hemisuccinate, 5 mM EDTA, 2 μM alprenolol, and 0.2 mg/ml FLAG peptide). Affinity-purified *β*1AR was further cleaned up by SEC, the monomeric receptor peak was pooled and concentrated, and aliquots (with 20% glycerol) were snap frozen in liquid N_2_ and stored at −80°C until use. Detergent-free high-density lipoprotein (HDL) particle (also referred to as nanodiscs) reconstitution of respective *β*-ARs (*β*1AR, *β*2AR, and *β*2AR-pP) were carried out following previously established procedures (Ahn et al., 2018; Staus et al., 2016). Receptor-containing HDLs were isolated from receptor-free HDL particles using anti–FLAG-M1 affinity chromatography followed by a further clean-up step by SEC.

### Membrane Purification

Membrane preparations from receptor-expressing cells were carried out following previously reported methods (Strachan et al., 2014) with minor modifications. Cells expressing *β*2AR-YFP or *β*2V2R were harvested in cold homogenization buffer (20 mM Tris-HCl, pH 7.4, 5 mM EDTA, 125 mM sucrose, containing 0.2 mM PMSF (phenylmethylsulfonyl fluoride) and EDTA-free protease inhibitor cocktail). Cell suspensions were dounce-homogenized and subjected to differential centrifugation to obtain microsomal crude membrane fractions. Isolated membranes were triturated, using a syringe with a 27-G needle, in cold membrane resuspension buffer (50 mM Tris-HCl, pH 7.4, 150 mM NaCl, 12.5 mM MgCl_2_, 2 mM EDTA, containing 10% glycerol and EDTA-free protease inhibitor cocktail). Aliquots of homogeneously resuspended membranes were snap frozen in liquid N_2_ and stored at −80°C until use.

### Radioligand Binding

**Fig. 1.**
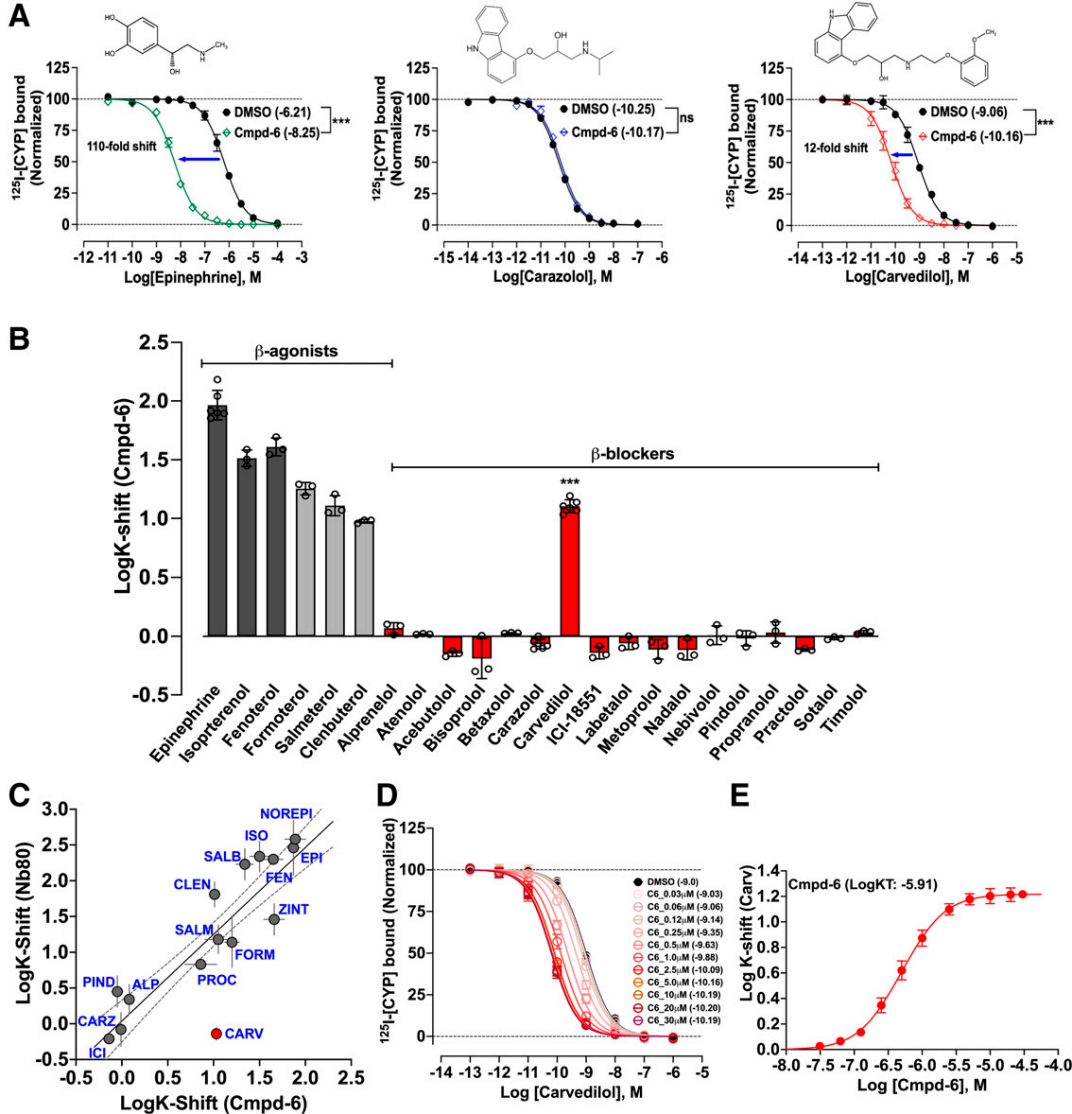
Cmpd-6 is positively cooperative with the *β*-blocker carvedilol. (A) Radioligand competition binding showing the displacement of ^125^I-CYP by the cold competitor’s epinephrine, carazolol, and carvedilol respectively at the *β*2AR in HDL. Data showing left-shifts in epinephrine (green) and carvedilol (red) competition curves indicate positive cooperativity with cmpd-6. Chemical structures of competing ligands are shown on top of respective data panel. Points on the curves represent normalized cpm values from three independent experiments ± S.D. (B) Bar graph showing cmpd-6–mediated affinity shifts (K-shifts), reported as the difference in LogIC50 (cmpd-6 versus DMSO) for a diverse panel of *β*-ligands: full agonists (gray), partial agonists (light gray), and anatgonists (red). Data represent shifts in LogIC50 (LogK-shifts) values derived from three to six independent experiments ± S.D. (C) Correlation plot showing comparison of affinity-shifts (LogK-shifts) for a panel of *β*-ligands mediated by Nb80 (Staus et al., 2016) and cmpd-6 (this study). Dashed lines around the line of correlation (solid gray) represent the 95% confidence interval. Carvedilol (red) is the one outlier ligand that is uniquely cooperative with only cmpd-6 but not Nb80. (D) Cmpd-6 dose response curves obtained by displacement of ^125^I-CYP by the cold carvedilol. (E) Curve showing shifts in LogIC50 (ΔLogIC50) of carvedilol mediated by cmpd-6 dose response; derived from data in (D). Points on the curves represent normalized cpm (D) and ΔLogIC50 (E) values from four independent experiments ± S.D. Statistical comparisons were done using one-way ANOVA with Bonferroni’s post hoc test. ***P < 0.001; ns, not significant.

Competition radioligand binding assays were performed at respective *β*-ARs (*β*1AR, *β*2AR, and *β*2AR-pP) reconstituted into HDL particles (or nanodiscs) or from isolated membrane preparations (*β*2AR-YFP or *β*2V2R) in assay buffer composed of 20 mM HEPES, pH 7.4, 100 mM NaCl, 0.2 mg/ml bovine serum albumin (BSA), and 0.18 mg/ml ascorbic acid (Ahn et al., 2018; Strachan et al., 2014). [^125^I]-Cyanopindolol (^125^I-CYP; 2200 Ci/mmol; PerkinElmer, Waltham, MA) was used at 60 pM and was competed with a serially diluted dose of unlabeled ligands without or with cmpd-6 or its analogs (20 µM). Competition bindings at *β*2AR-pP were carried out in the absence of presence of cmpd-6 (1 µM) along with either Gs-*αβγ* heterotrimer (GsHet; 5 nM) or *β*-arrestin1 (*β*arr1; 250 nM). All binding assays were carried out until equilibrium at room temperature in a final reaction volume of 200 µl. Equilibrated binding reactions were harvested onto glass-fiber filters (GF/B) and presoaked with 0.3% (vol/vol) polyethyleneimine in deionized water, using a 96-well Brandel harvester (Brandel, Gaithersburg, MD). The filters were rapidly washed with 10 ml cold wash buffer (20 mM HEPES, pH 7.4, 100 mM NaCl), and the bound ^125^I-CYP was measured using a 2470 Wizard^2^ 2-Detector Gamma Counter (PerkinElmer). Competition binding data were analyzed in GraphPad Prism 9.0 (GraphPad Software, La Jolla, CA) using a nonlinear regression curve fit and the one-site-Fit LogIC50 equation to derive the estimates of equilibrium binding constant (Kd) for respective conditions, and normalized cpm values were plotted as means ± S.D. The titration curves representing the change of carvedilol binding affinity with increasing concentrations of PAM (data shown in Figs. 1D and 5D; Supplemental Fig. 2), were fitted using the following equation:

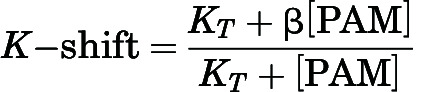


where K-shift indicates the ratio of carvedilol dissociation constants measured in the absence and presence of allosteric modulator, K_T_ is the dissociation constant of PAM for receptor binding, and *β* gauges the cooperativity factor between carvedilol and PAM binding. To estimate K_T_ and *β*, the data were analyzed by nonlinear regression with a user-defined version of the above equation added to the model library of GraphPad Prism.

[^3^H]-R/S-Carvedilol (^3^H-Carv, 80 Ci/mmol; American Radiolabeled Chemicals, Inc., St. Louis, MO) binding was performed by a scintillation proximity assay (SPA) using FLAG-tagged yttrium silicate (YSi) SPA microbeads (PerkinElmer). Nanodisc-reconstituted *β*2AR-pP was incubated, in the aforementioned assay buffer, for 90 minutes at room temperature with ^3^H-Carv (1 nM) in the presence of DMSO (0.2%) or cmpd-6 (20 µM), GsHet (100 nM), and *β*arr1 (1 μM). Nonspecific bindings were assessed by using saturating concentration of cold propranolol (20 µM). Receptor was captured on YSi beads via FLAG-tag, and bound radioligand was detected using a Wallac 1450 microBeta scintillation counter (PerkinElmer). Bound specific cpm was expressed as ligand binding in fmol and plotted as bar graphs in GraphPad Prism.

Guanosine 5*γ*-*O*-(3-[^35^S]thio)triphosphate (^35^S-GTP*γ*S; 1250 Ci/mmol; PerkinElmer) binding to GsHet was performed using SPA in assay buffer containing 20 mM HEPES, pH 7.4, 100 mM NaCl, 10 mM MgCl_2_, 10 μM GDP, 0.2 mg/ml BSA, and 0.18 mg/ml ascorbic acid. Nanodisc-reconstituted *β*2ARs were incubated with GsHet (100 nM) in the absence (DMSO) or presence of saturating concentration of cmpd-6 (20 µM) or A9 (20 µM), Nb6B9 (1 µM), and stimulated with epinephrine (10 µM) or ICI-118551 (10 µM). ^35^S-GTP*γ*S was used at 200 pM, and binding reactions were carried out for 60 minutes at room temperature. Basal ^35^S-GTP*γ*S binding to Gs protein was determined in absence of agonist stimulation, and nonspecific binding was determined by including nonradioactive GTP*γ*S (20 µM). After incubations, the *β*2AR-Gs complexes were captured on YSi beads. Bound radioligand was detected using a Wallac 1450 microBeta scintillation counter (PerkinElemer). Specific ^35^S-GTP*γ*S binding for respective conditions was normalized to no ligand stimulation, expressed as fold over basal, and plotted as bar graphs in GraphPad Prism.

### Measurements of cAMP Generation

cAMP production was monitored at endogenous *β*2AR in HEK293 cells stably expressing the plasmid for GloSensor luciferase enzyme (Promega), a chemiluminescence-based cAMP biosensor. Cells were plated in 96-well, white clear-bottom plates at a density of ∼80,000 cells per well, and on the following day, chemiluminescence signals generated by the GloSensor luciferase were read using a CLARIOstar microplate reader (BMG Labtech, Cary, NC) as previously described (Ahn et al., 2018). Prior to ligand stimulations, cells were treated with GloSensor reagent and incubated at 27 degrees for 1 hour to allow equilibration. Cells were then treated with either cmpd-6 (20 μM) or a vehicle control (DMSO) diluted in Hanks’ balanced salt solution (HBSS) supplemented with 20 mM HEPES, 0.05% BSA, and 3-isobutyl-1-methylxanthine at a final concentration of 100 μM. For cAMP generation by ligands tested in *agonist* mode, a serial dilution of either epinephrine or carvedilol diluted in HBSS supplemented with 20 mM HEPES was then added to cells, and changes in luminescence were read at various time points ranging from 5 to 35 minutes after addition of orthosteric ligand. To assess blockade of agonist-stimulated cAMP generation, respective *β*-blockers (carvedilol or metoprolol) were tested in *antagonist* mode. A serial dilution of these *β*-blockers was first added to cells and incubated at 27 degrees for ∼10 minutes followed by epinephrine at a final concentration of 1 μM diluted in HBSS supplemented with 20 mM HEPES was added to cells. Changes in chemiluminescence were then read at various time points after stimulation. Concentration response curves were then generated by plotting normalized chemiluminescence values and were analyzed in GraphPad Prism using nonlinear regression analysis and log (inhibitor) versus response function.

### Cell Surface ELISA

U2OS cells stably expressing HA-tagged *β*2V2R (a chimeric *β*2AR with C-terminal tail of V2R and *β*-arrestin2–GFP) (Oakley et al., 1999) were used to assess the extent of receptor internalization in intact cells using cell surface receptor ELISA. Cells were cultured for overnight in 48-well tissue culture plates and subsequently serum-starved overnight in DMEM containing 0.1% IgG- and protease-free BSA (Jackson ImmunuResearch Laboratories, Inc., West Grove, PA), 20 mM HEPES, pH 7.4, and 1× GlutaMax (Invitrogen). After serum starvation, cells were stimulated for 16 hours with a serial dilution of respective orthosteric ligands (epinephrine, carvedilol, or ICI-118551) in the absence (DMSO) or presence of cmpd-6 (5 μM). Thereafter, stimulated cells were fixed in freshly prepared 3.6% paraformaldehyde in HBSS for 30 minutes on ice, quenched with Tris-HCl (250 mM) and 0.3% H_2_O_2_, washed, and blocked with 3% nonfat dry milk in HBSS. Cell-surface *β*2V2R were labeled using horseradish peroxidase–conjugated anti–HA-tag antibody (Sigma, clone 3F10 used at 1:2500 dilution), developed using 150 μl ultraTMB substrate (Thermo Fisher Scientific, Waltham, MA), and quenched with 150 μl acidified HBSS (0.2 N H_2_SO_4_). A 100 μl aliquot of quenched reaction from each condition was transferred to a 96-well plate for colorimetric readings at 450 nm using a CLARIOstar microplate reader (BMG Labtech). Primary assay plates were gently washed with deionized water and stained with 0.2% Janus Green B in HBSS to estimate total cells per well (Raspotnig et al., 1999) for data normalizations. After staining, cells were gently washed with deionized water, and accumulated Janus Green B stain was extracted in 300 μl acidified HBSS (0.5 N HCl). A 100 μl aliquot of the extracted whole cell staining from each condition was then transferred to a 96-well plate, and colorimetric readings were taken at 595 nm. Absorbance values for cell surface receptor (450 nm) for each condition were normalized with corresponding whole cell staining (595 nm) and analyzed in GraphPad Prism using nonlinear regression analysis and log (ligand) versus response function to derive estimates of EC50 values for respective ligands in the absence (DMSO) or presence of cmpd-6.

### Measurement of *β*2AR Endocytosis

*β*2AR endocytosis was monitored in a high-throughput way using a chemiluminescence-based enzyme fragment complementation assay (Eurofins) according to the manufacturer’s recommendations, with minor modifications. HEK293 cells, stably expressing the Enzyme Acceptor–tagged *β*2AR and endosome-localized ProLink-tethered protein, were plated in 96-well white, clear-bottomed plates at a density of ∼80,000 cells per well. On the following day, cells were treated with DMSO or cmpd-6 at 10–30 µM for 10 minutes and then stimulated with a serial dilution of carvedilol for 16 hours to accumulate signals over that time. The extent of *β*2AR trafficking to endosomes was measured as chemiluminescence signals resulting from the complementation of *β*-galactosidase fragments (Enzyme Acceptor and ProLink) at endosomes and was detected on a CLARIOstar plate reader (BMG Labtech) using a detection kit (Eurofins).

### Extracellular Signal-Regulated Kinase Phosphorylation

HEK293 cells stably expressing the *β*2AR were plated on 6-well plates at a density to achieve ∼50–70% confluency prior to serum starvation on the following day. Serum-free medium was prepared by supplementing MEM with 0.1% BSA, 10 mM HEPES (pH 7.4), and 1× penicillin/streptomycin into standard minimum Eagle’s growth medium. After an overnight serum starvation, cells were pretreated with cmpd-6 at 5 µM, stimulated with a serial dilution of carvedilol for 5 minutes, and solubilized by directly adding 2× SDS-sample buffer. After sonication with a microtip for 15 seconds, equal amounts of cellular extracts were separated on 4–20% Tris-glycine polyacrylamide gels (Invitrogen), and resolved proteins were transferred onto nitrocellulose membranes (Bio-Rad, Hercules, CA) for immunoblotting. Detection of total and phosphorylated extracellular signal-regulated kinase (ERK) 1/2 on immunoblots were carried out with rabbit polyclonal anti–phospho-p44/42 mitogen-activated protein kinase (used at 1:2000 dilution; Cell Signaling Technology, Danvers, MA) and anti–mitogen-activated protein kinase 1/2 (MilliporeSigma, used at 1:8000 dilution) antibodies. Chemiluminescence signals were developed using the SuperSignal West Pico reagent (Thermo Fisher Scientific), visualized using a ChemiDoc imaging system (Bio-Rad), quantified by a densitometry software, Image Laboratory (Bio-Rad), and analyzed using GraphPad Prism 9.0.

### Confocal Imaging

HEK293 cells stably expressing *β*2AR-YFP were plated on poly-d-lysine–coated glass-bottom dishes (MatTek, Ashland, MA). The next day, cells were serum-starved overnight in MEM supplemented with 20 mM HEPES (pH7.4) and 1 mg/ml BSA. Cells were loaded with Lysotracker Red dye as per manufacturer’s instructions (Invitrogen) for 30 minutes followed by an additional 30 minutes of stimulation with carvedilol (10 nM) or ICI-118551 (10 nM) in the absence (DMSO) or presence of cmpd-6 (5 μM). After ligand stimulations, cells were fixed for 30 minutes at room temperature in a freshly prepared 3.6% paraformaldehyde solution in HBSS, washed, and imaged in FluroBrite DMEM (Invitrogen). Samples were imaged using a Ziess LSM 710 confocal microscope equipped with the Yokogawa CSU-X1 spinning disc system and an Evolve 512 EMCCD camera (Photometrics, Tucson, AZ). *β*2AR-YFP (green) was illuminated using a 488 nm laser, and stained lysosomes (red) were imaged using 561 nm (for Lysotracker Red) laser. Fluorescent images were captured using both 63× and 100× oil objectives. Captured images were deconvoluted using no neighbor deconvolution to improve signal-to-noise ratio for quantitative analysis. Images were analyzed in 3i’s SlideBook 6 program using the colocalization analysis tool. Background pixels were eliminated using Costes’ automatic thresholding, and pixels with overlapping red and green intensity were counted as collocated pixels (Costes et al., 2004). Each experimental condition surveyed four to seven independent images with each image containing 15 to 20 cells. Fraction of collocated pixels were determined for each image by dividing the collocated pixel count by the total number of green (*β*2AR-YFP) and red (lysosomes) pixels. The resultant fractions of collocated pixels were normalized to the number of cells within an image to yield the colocalization indices for respective treatments and plotted using GraphPad Prism.

### Data Analysis and Statistics

Data analysis and plotting was performed using GraphPad Prism 9.0 and Microsoft Excel. Statistical comparisons were made using unpaired *t* test, one-way or two-way ANOVA with Bonferroni’s multiple comparisons post hoc tests. Experimental values are expressed as means ± SD. Differences in the mean values were considered to be significant at *P* < 0.05.

## Results

### At the *β*2AR, Cmpd-6 is Uniquely Cooperative with Carvedilol Among *β*-Blockers

Cmpd-6 is a recently identified *β*2AR-specific PAM that selectively shows positive cooperativity with *β*-agonists but not antagonists at the *β*2AR (Ahn et al., 2018; Liu et al., 2019). Upon further examination with a structurally and pharmacologically diverse panel of *β*-AR ligands (Supplemental Fig. 1), we found that cmpd-6 is uniquely, and quite unexpectedly, cooperative with the *β*-blocker carvedilol (Fig. 1, A and B). Consistent with the previous report (Ahn et al., 2018), cmpd-6 shifts the ^125^I-CYP displacement binding curve of the full agonist epinephrine to the left by ∼2-log to higher affinity [Fig. 1A; epinephrine-DMSO LogIC50 = −6.211, 95% confidence interval (CI) (−6.245 to −6.177); epinephrine+cmpd-6 LogIC50 = −8.254, 95% CI (−8.288 to −8.220)]. As would be anticipated for an antagonist, there was no effect of cmpd-6 on the binding curve of carazolol, a close structural relative of carvedilol that shares the same carbazole head group (Fig. 1, A and B). However, surprisingly, the competition curve of the *antagonist* carvedilol was left-shifted by ∼1.2 log in presence of cmpd-6 [Fig. 1A; carvedilol-DMSO LogIC50 = −9.062, 95% CI (−9.093 to −9.031); carvedilol+cmpd-6 LogIC50 = −10.16, 95% CI (−10.20 to −10.13)]. Among a total of 17 *β*-blockers tested (Fig. 1B) this phenomenon was unique only to carvedilol. Furthermore, as shown in Fig. 1C, across a diverse set of *β*-ligands, there was a strong positive correlation (R^2^ = 0.83 without carvedilol) between cooperative effects of cmpd-6 with those of another PAM for the *β*2AR, nanobody-80 (Nb80), which pharmacologically behaves as a Gs mimic (DeVree et al., 2016; Rasmussen et al., 2011a; Staus et al., 2016). The one glaring exception from this correlation was carvedilol, which further underscores the unique positive cooperativity between cmpd-6 and carvedilol. We next determined the binding affinity of cmpd-6 for the *β*2AR in the presence of carvedilol in competition ligand binding assays (Fig. 1, D and E). Cmpd-6, in a dose-dependent manner, resulted in progressive left-shifts of ^125^I-CYP displacement curves by carvedilol (Fig. 1D). Titrations of cmpd-6 resulted in nested leftward curve shifts for carvedilol (Fig. 1D) and by plotting the difference in carvedilols' LogIC50 versus dose of [cmpd-6] we determined that cmpd-6 binds the receptor with ∼1.2 × 10^−6^ M affinity in the presence of carvedilol [Fig. 1E; cmpd-6 LogK_T_ = −5.913, 95% CI (−5.969 to −5.852)]. This binding affinity value of cmpd-6 is ∼4.3-fold stronger than its binding affinity for the agonist-occupied *β*2AR as previously determined by isothermal titration calorimetry (Ahn et al., 2018). Notably, although the PAM activity of cmpd-6 with respect to *agonists* is highly receptor subtype selective (*β*2AR≫*β*1AR), we identify that its unique cooperativity with carvedilol is also conserved at the *β*1AR (Supplemental Fig. 2, A and B). The allosteric effect of cmpd-6 at the *β*1AR was saturable with a maximal curve shift of ∼0.9 log [Supplemental Fig. 2A; DMSO LogIC50 = −8.849, 95% CI (−8.885 to −8.813); cmpd-6 LogIC50 = −9.731, 95% CI (−9.770 to −9.693)]. The binding affinity of cmpd-6 was determined to be ∼1.7 × 10^−5^ M [Supplemental Fig. 2B; cmpd-6 LogK_T_ = −4.766, 95% CI (−5.146 to −3.951)]. Taken together, these data identify a unique, nonreceptor subtype-specific cooperativity between cmpd-6 and carvedilol among a large number of *β*-blockers. A detailed characterization of the allosteric effects of cmpd-6 on carvedilol-mediated *β*1AR signaling is reported in the accompanying manuscript by Wang et al. (2021).

### Cmpd-6 Facilitates *β*-Arrestin1–Induced High-Affinity Binding of Carvedilol to the *β*2AR

**Fig. 2.**
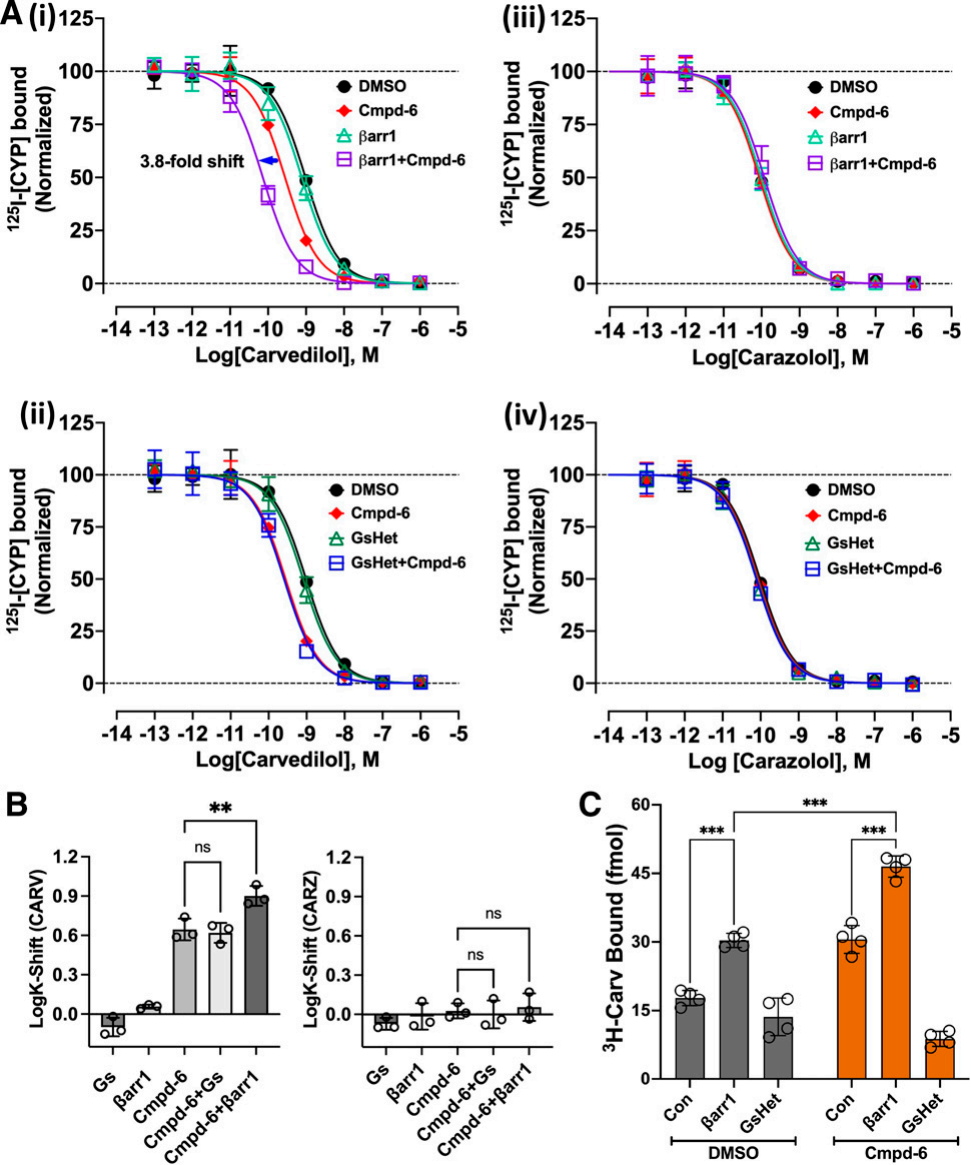
Cmpd-6 and *β*-arrestin1–mediated high-affinity binding of carvedilol. (A) Radioligand competition binding showing the displacement of ^125^I-CYP ([^125^I]-Cyanopindolol) by the cold competitors carvedilol [(i) and (ii)] and carazolol [(iii) and (iv)], respectively, at the *β*2V2RpP in HDL. Cmpd-6 is shown to display cooperative effects with *β*arr1 (i), but not with Gs (ii), in promoting high-affinity left-shifts of carvedilol competition curves. No such cooperativity is observed in carazolol competition curves. (B) Bar graph showing comparison of CARV (carvedilol) and CARZ (carazolol) affinity-shifts (LogK-shifts) mediated by cmpd-6. Data shown in (A) and (B) represent values obtained from three independent experiments ± S.D. (C) Bar graph showing the direct high-affinity binding of ^3^H-Carv to *β*2ARpP in HDL. Compared with DMSO control (Con), cmpd-6 alone and together with *β*arr1, but not Gs, is shown to potentiate ^3^H-Carv binding. Data shown in the bar graphs represent mean receptor binding values obtained from four independent experiments ± S.D. Statistical comparisons were made by two-way ANOVA followed by Bonferroni’a post hoc test. **P < 0.01; ***P < 0.001; ns, not significant.

Carvedilol is a therapeutic *β*-blocker with a unique bias toward activating *β*-arrestin signaling. Thus, allosteric modulation of carvedilol function might be of therapeutic importance. Accordingly, we assessed the cooperative effects of cmpd-6 on transducer (Gs or *β*-arrestin1) coupling to the *β*2AR. We monitored either carvedilol competition radioligand binding (Fig. 2, A and B) or direct binding of radiolabeled carvedilol (^3^H-Carv) to the *β*2AR (Fig. 2C) in the absence (DMSO) or presence of cmpd-6 together with either transducer. To facilitate binding of *β*arr1 to the receptor, a synthetically phosphorylated C-tail of V2R was ligated in vitro at the C-terminal end of the *β*2AR (*β*2V2R-pP) using the sortase enzyme as previously described (Staus et al., 2018), and the phospho-peptide ligated receptor was reconstituted into detergent-free HDL particles. Cmpd-6 showed positive cooperativity with *β*arr1 [Fig. 2A(i); cmpd-6 LogIC50 = −9.554, 95% CI (−9.626 to −9.482); *β*arr1+cmpd-6 LogIC50 = −10.14, 95% CI (−10.20 to −10.07)] but not with Gs [Fig. 2A(ii); GsHet+cmpd-6 LogIC50 = −9.594, 95% CI (−9.714 to −9.475)] and enhanced the ability of carvedilol [but not carazolol; Fig. 2A(iii)–(iv)] to compete against the radiolabeled tracer ^125^I-CYP binding to the receptor. Remarkably, the combined application of cmpd-6 and *β*arr1 (but not Gs) resulted in a further high-affinity left-shift of the competition curve compared with that obtained using respective transducers tested individually (Fig. 2B). Additionally, and consistent with the competition binding, we show that cmpd-6 in the presence of *β*arr1 (but not Gs) substantially increases the direct high-affinity binding of ^3^H-Carv to the *β*2AR (Fig. 2C).

### Cmpd-6 Positively Modulates Carvedilol-Stimulated Cellular *β*2AR Functions

**Fig. 3.**
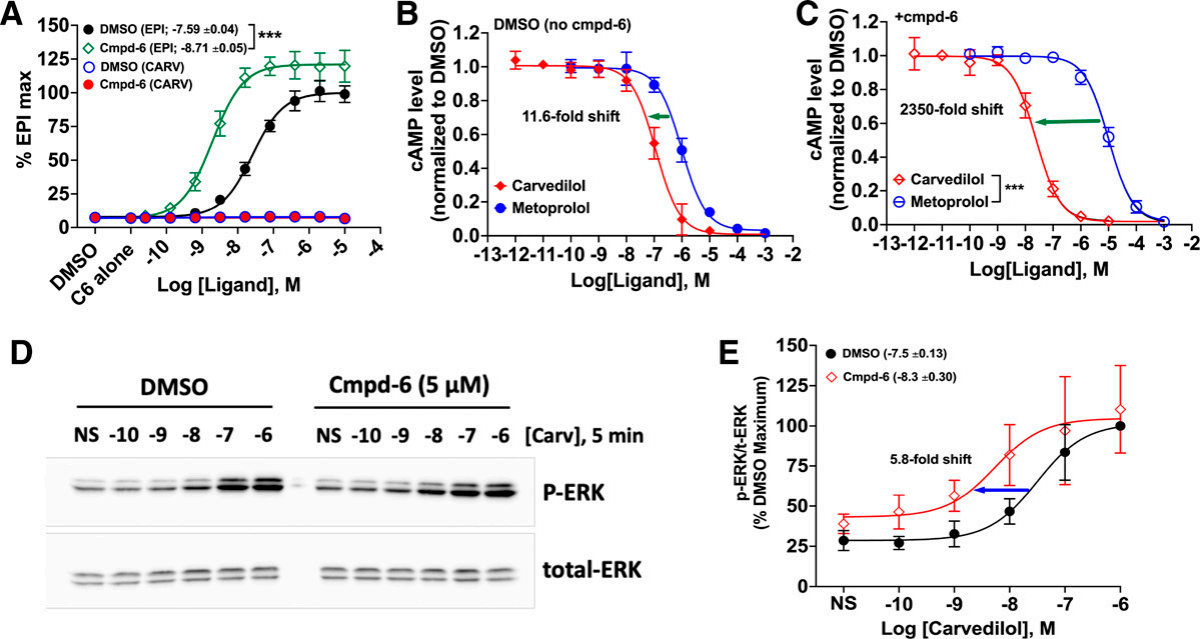
Cmpd-6 augments cellular activity of carvedilol at the *β*2AR. (A) HEK293 cells stably expressing GloSensor were pretreated with either vehicle (DMSO) alone or cmpd-6 (C6) (30 µM) for 15–20 minutes. The extent of cAMP generation by endogenously expressed *β*2AR was subsequently measured after stimulation of the cells with either epinephrine (EPI) or carvedilol (CARV) for 5–10 minutes in a dose-dependent manner. Values were normalized to the maximal level of EPI-induced activity in the vehicle (0.3% DMSO) control, expressed as a percentage, and represent means ± S.D. (B, C) Cells were pretreated with either vehicle DMSO alone (B) or cmpd-6 (C) for 15–20 minutes, and inhibition of 1 µM EPI-stimulated cAMP generation was monitored in the presence of a dose of either carvedilol or metoprolol. Values were normalized to the uninhibited EPI signal in the DMSO control. Dose-dependent curve fits were generated with data points obtained from three or four independent experiments done in duplicate. (D) HEK293 cells stably expressing the *β*2AR were serum-starved overnight and subsequently pretreated with either vehicle (DMSO) alone or cmpd-6 at 5 µM for 15–20 minutes. The cells were either non-stimulated (NS) or stimulated with carvedilol for 5 minutes in a dose-dependent manner. ERK phosphorylation (p-ERK) and total ERK expression (t-ERK) in each sample were visualized by immunoblotting as described. (E) Each of the p-ERK and t-ERK bands in the immunoblot was quantified as described, and the extent of ERK phosphorylation was determined through dividing the p-ERK signal by the t-ERK. Each data point was expressed as percent of the maximal response in the vehicle-treated control cells and represents the mean ± S.D. from five independent experiments. Dose-response curves and EC50 values between vehicle (DMSO)– and cmpd-6–treated samples were obtained by using GraphPad Prism. Statistical significance for the difference in LogKi values (carvedilol versus metoprolol) between vehicle (DMSO)– and cmpd-6–treated curve fits (*P* < 0.001) was determined by two-way ANOVA. ***P < 0.001.

To determine the cellular implications of the allosteric effect of cmpd-6 on carvedilol-mediated signaling at the *β*2AR, we performed a series of cell-based assays. We first tested the activation of Gs by monitoring cAMP generation. When used in an *agonist mode*, carvedilol alone and together with cmpd-6 did not stimulate any detectable levels of cAMP production, unlike the robust response to the agonist epinephrine stimulation, which was augmented by cmpd-6 [Fig. 3A; epinephrine LogEC50 = −7.587, 95% CI (−7.664 to −7.510); epinephrine+cmpd-6 LogEC50 = −8.709, 95% CI (−8.807 to −8.611)]. We next tested carvedilol in an *antagonist mode*, essentially evaluating its ability to block the agonist epinephrine-stimulated cAMP responses in comparison with a control ligand, metoprolol, with which cmpd-6 had no positive cooperativity as shown in competition binding (Fig. 1B). Interestingly, in comparison with metoprolol, we found that cmpd-6 substantially augments the blockade of agonist-stimulated cAMP generation by carvedilol [Fig. 3C; +cmpd-6, carvedilol LogKi = −10.33, 95% CI (−10.44 to −10.22]; metoprolol LogKi = −7.703, 95% CI (−7.788 to −7.618)]. Compared with metoprolol-mediated inhibition of epinephrine-stimulated cAMP generation, the presence of cmpd-6 remarkably led to ∼2350-fold leftward shift of the carvedilol dose-dependent inhibitory curve (Fig. 3C versus ∼11-fold shift without cmpd-6, Fig. 3A). Furthermore, this measure of fold-shift in inhibition of cAMP with carvedilol must be underestimated since cmpd-6 also potentiates the epinephrine-stimulated response. These results suggest that the allosteric modulation of carvedilol by cmpd-6 enhances the cellular *β*-blockade potency of the ligand. We then tested the effect of cmpd-6 on carvedilol-stimulated ERK phosphorylation downstream of the *β*2AR, which has been shown to be *β*-arrestin–dependent (Wisler et al., 2007). In HEK cells stably overexpressing the *β*2AR, cmpd-6 substantially enhanced (by ∼5.8-fold) the potency of carvedilol-stimulated ERK phosphorylation [Fig. 3, D and E; DMSO LogEC50 = −7.504; 95% CI (−7.774 to −7.234); +cmpd-6 LogEC50 = −8.268, 95% CI (−8.899 to −7.638)].

### Cmpd-6 Enhances Carvedilol-Stimulated Internalization of the *β*2AR

To further evaluate the cellular effects of this unique positive cooperativity, we tested the role of cmpd-6 on carvedilol-mediated receptor internalization, a function attributable to *β*-arrestins. To this end we employed U2OS and HEK293 cells stably expressing the chimeric *β*2V2R (Oakley et al., 1999) or the YFP-tagged *β*2AR, respectively (Han et al., 2012). These recombinant versions of the human *β*2AR were pharmacologically validated by competition ligand binding using membrane preparations from respective cell lines (Supplemental Fig. 3). Consistent with our in vitro binding data (Fig. 1A), both versions of the *β*2AR, in cell membrane preps, retained the cooperative effect of cmpd-6 with epinephrine and carvedilol but not with carazolol. The affinity-shifts for carvedilol elicited by cmpd-6 at the *β*2V2R [Supplemental Fig. 3C; DMSO LogIC50 = −9.019, 95% CI (−9.043 to −8.996); cmpd-6 LogIC50 = −9.959, 95% CI (−9.999 to −9.919)] and *β*2AR-YFP [Supplemental Fig. 3F; DMSO LogIC50 = −9.086, 95% CI (−9.128 to −9.043); cmpd-6 LogIC50 = −9.903, 95% CI (−9.936 to −9.870)] were conserved and comparable to the results obtained with the wild-type *β*2AR (Fig. 1A).

**Fig. 4.**
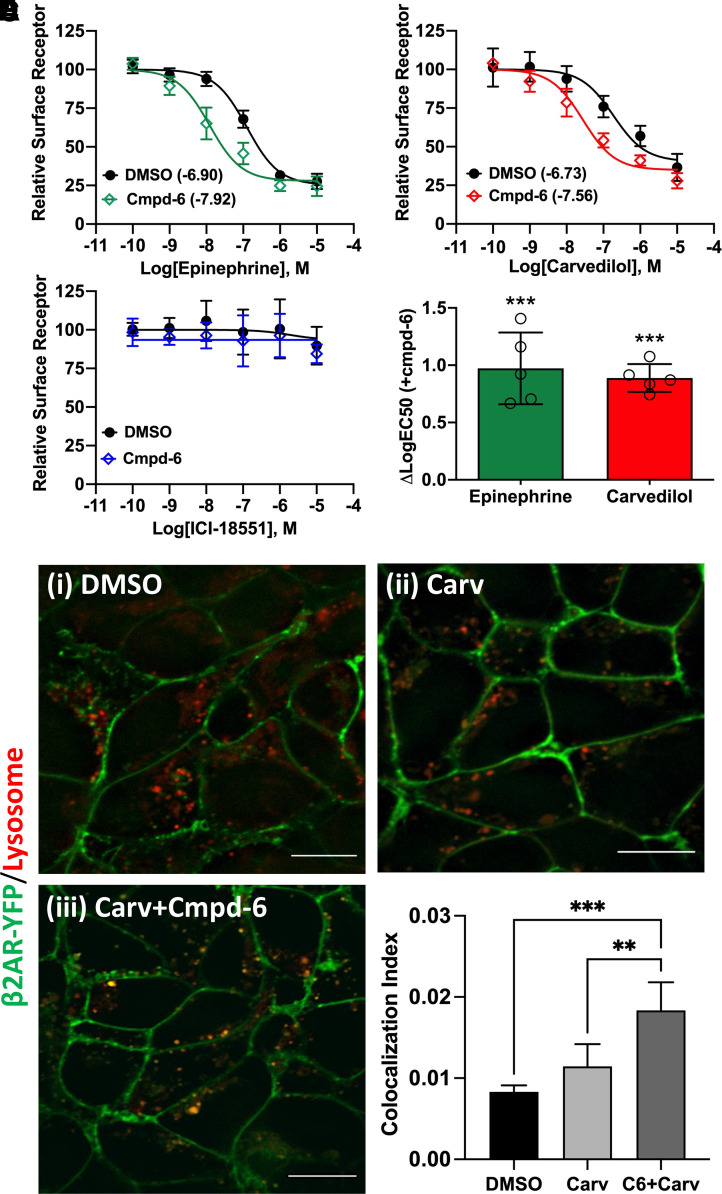
Cmpd-6 augments carvedilol-stimulated *β*2AR internalization. (A–C) Cell surface ELISA showing the cooperative effects of cmpd-6 on loss of cell-surface *β*2AR after a dose response of epinephrine (A), carvedilol (B), and ICI-118551 (C); normalized mean ± S.D, *n* = 5. (D) Bar graph showing mean difference in LogEC50 (versus DMSO control) for epinephrine (green, *n* = 5) and carvedilol (red, *n* = 5) in presence of cmpd-6. (E) Representative confocal images showing cmpd-6–mediated potentiation of lysosomal targeting of the *β*2AR upon stimulation with carvedilol (Carv). Scale bar on respective images is 10 μm. (F) Bar graph showing quantification of *β*2AR-YFP (green) colocalization with lysosomes (red); expressed as colocalization index ± S.D. as described in *Materials and Methods*. Statistical comparisons were made using one-way ANOVA with Bonferroni’s post hoc tests. **P < 0.01; ***P < 0.001.

We employed multiple orthogonal assay formats to evaluate carvedilol-stimulated receptor endocytosis in the absence (DMSO) or presence of cmpd-6: by measuring the loss of cell-surface *β*2V2R by ELISA (Fig. 4, A–D), by confocal imaging of *β*2AR-YFP trafficking to lysosomes (Fig. 4, E and F), and by DiscoverX (Fremont, CA) enzyme complementation assay for total receptor endocytosis (Supplemental Fig. 4). Collectively, in all these aforementioned cellular assays, the effect of carvedilol together with cmpd-6 was significantly greater than that mediated by carvedilol alone. We found that cmpd-6, as with the agonist epinephrine [Fig. 4A; DMSO LogEC50 = −6.901; 95% CI (−7.008 to −6.795); cmpd-6 LogEC50 = −7.916, 95% CI (−8.101 to −7.730)], enhanced the potency (by ∼6.8-fold change in EC50) of carvedilol-stimulated endocytosis of the receptor as assessed by cell surface ELISA [Fig. 4B; DMSO LogEC50 = −6.731, 95% CI (−7.018 to −6.445); cmpd-6 LogEC50 = −7.561, 95% CI (−7.764 to −7.358)], as well as by total receptor endocytosis (Supplemental Fig. 4). However, consistent with the binding data (Fig. 1B), no such cooperative effect of cmpd-6 on endocytosis of the receptor was observed in cells treated with the inverse agonist ICI-118551 (Fig. 4C). This further corroborates the unique positive cooperativity of cmpd-6 with the β-blocker carvedilol. Of note, even more effectively than agonists, carvedilol has been reported to target the β2AR to lysosomal compartments (Han et al., 2012). Thus, we tested the cooperative effect of cmpd-6 on the carvedilol-stimulated lysosomal targeting of the β2AR-YFP stably expressed in HEK-293 cells. After ligand stimulation, the colocalization of β2AR-YFP with the lysosomal marker dye (Lysotracker Red) was visualized and quantified (Fig. 4, E and F). Interestingly, compared with DMSO control, cmpd-6 substantially augmented carvedilol-mediated lysosomal targeting of the β2AR. These data further underscore the signaling impact of the cooperative effects of cmpd-6 on carvedilol-mediated cellular functions. In essence, cmpd-6 not only enhances the cellular β-blockade potency of carvedilol but also positively augments β-arrestin–mediated cellular signaling emanating from the carvedilol-occupied β2AR.

### Development of a Carvedilol-Specific Allosteric Modulator

**Fig. 5.**
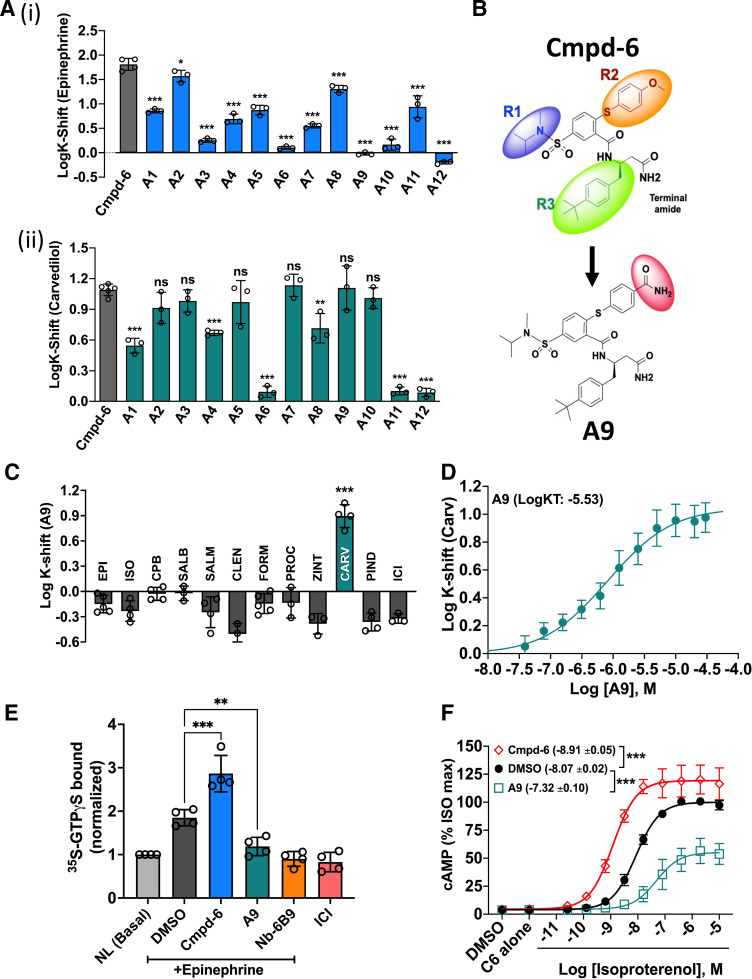
Cmpd-6 analog A9 displays carvedilol-specific cooperativity at the *β*2AR. (A) Bar graph showing affinity shifts (LogK-shifts) of epinephrine (i) and carvedilol (ii) mediated by cmpd-6 (gray bars) or its analogs (A1–A12, colored bars) compared with DMSO control. (B) Chemical structures of cmpd-6 and its analog A9 highlighting the modified R2 moiety in the analog. (C) Bar graph showing the allosteric cooperative effects of A9 on affinity shifts of a panel of *β*-ligands (agonists and antagonists). A9 shows positive cooperativity only with carvedilol compared with other ligands tested. Bar graphs in (A) and (C) show mean LogK-shifts ± S.D. obtained from three to five independent experiments. (D) Curve showing affinity shifts in LogIC50 (LogK-Shift) for carvedilol mediated by a dose of A9. Points on the curves represent LogK-Shift values derived from three independent competition binding experiments ± S.D. (E) Bar graph showing binding of ^35^S-GTP*γ*S to heterotrimeric Gs either under basal, no ligand (NL) condition or after epinephrine stimulation of the *β*2AR in HDL. Data for respective conditions (*n* = 4) are normalized to basal (unstimulated) binding of ^35^S-GTP*γ*S. (F) Curves showing the effect of cmpd-6 and A9 on isoproterenol (ISO)–stimulated cAMP generation. Cells were pretreated for 15–20 minutes with either cmpd-6 or A9 (30 µM) and then stimulated with a dose of ISO. The amount of cAMP production by endogenously expressed *β*2AR was measured 10 minutes after ISO stimulation. Curve fits were generated in GraphPad Prism with data points obtained from five independent experiments done in duplicate. Each data point was normalized to the maximal level of ISO-induced activity in the vehicle (DMSO) control, expressed as a percentage, and represents mean ± S.D. Statistical comparisons were made using one-way ANOVA with Bonferroni’s post hoc tests. *P < 0.05; **P < 0.01; ***P < 0.001; ns, not significant.

Although cmpd-6 potentiates the *β*-arrestin–biased agonism of carvedilol, it is also a PAM that is positively cooperative with agonists at the *β*2AR in an unbiased manner (Ahn et al., 2018). We thus set out to test a set of chemically modified analogs of cmpd-6 (A1–A12) with the hopes to identify molecules that would retain the positive allosteric cooperativity with carvedilol while losing the PAM activity with *β*-agonists. Such modified analogs would not only be of potential therapeutic value but might also pave the way for the development of novel and biased allosteric drugs targeting other GPCRs. Among the several cmpd-6 analogs whose structures we have previously reported (Liu et al., 2019), we identified the analog A9 [Fig. 5A(i)–(ii)] to display the desired allosteric cooperative properties. Structurally, A9 differs from its parent cmpd-6 in carrying a terminal amide group at the R2 moiety (Fig. 5B). Although A9 retains its positive cooperativity with carvedilol comparable to the level obtained with the parent cmpd-6, it shows absolutely no PAM activity with agonists including epinephrine (Fig. 5A(i); Fig. 5C). The allosteric effect of A9 to affinity-shift carvedilol competition curves to the left is saturable and the analog has ∼2.9 × 10^−6^ M affinity for the carvedilol-bound *β*2AR [Fig. 5D; A9 LogK_T_ = −5.533, 95% CI (−5.737 to −5.247)], which is comparable to the ∼1.2 × 10^−6^ M affinity determined for cmpd-6 [Fig. 1E; cmpd-6 LogK_T_ = −5.913, 95% CI (−5.969 to −5.852)]. Interestingly, unlike the PAM activity of cmpd-6 in potentiating *β*-agonist–stimulated responses, A9 remarkably shows a robust NAM activity for agonist-mediated *β*2AR functions. Whereas cmpd-6 further augments the activation of heterotrimeric Gs by agonists, A9 markedly blocks the agonist-stimulated activation of Gs both in vitro and in cells, as shown by A9-mediated reduction in ^35^S-GTP*γ*S binding to Gs in vitro (Fig. 5E). The binding of non-hydrolyzable ^35^S-GTP*γ*S to Gs is driven by physical coupling of Gs to the *β*2AR and is increased in response to stimulation by the agonist epinephrine. However, in the presence of the antagonist/inverse agonist ICI-118551, no increase in ^35^S-GTPyS binding to Gs was observed. Additionally, and as expected, competition with Nb6B9 (an affinity-matured version of the Gs mimic nanobody, Nb80), which shares the transducer binding site (Ring et al., 2013), also reduces ^35^S-GTP*γ*S binding to Gs (Fig. 5E). Furthermore, in cells, A9 (unlike cmpd-6) also functions as a classic NAM inhibiting agonist isoproterenol-stimulated cAMP generation downstream of the activated *β*2AR [Fig. 5F; DMSO LogEC50 = −8.073, 95% CI (−8.122 to −8.023); cmpd-6 LogEC50 = −8.914, 95% CI (−9.021 to −8.807); A9 LogEC50 = −7.320, 95% CI (−7.524 to −7.115)].

## Discussion

In this study we report on a unique and unexpected pharmacological cooperativity between the recently discovered PAM of the *β*2AR, cmpd-6, and the Food and Drug Administration–approved *β*-blocker, carvedilol. Our findings unveil cmpd-6 as a positive allosteric modulator for the pharmacological activity of carvedilol at the *β*2AR as well as the closely related subtype, *β*1AR. Remarkably, the cooperativity of cmpd-6 is highly specific to carvedilol among a diverse array of known *β*-blockers tested in this study. Using orthogonal experimental approaches, both in vitro and in cultured cells, we demonstrate that cmpd-6 augments the binding affinity of carvedilol for both *β*1AR and *β*2AR, the potency of carvedilol’s *β*-blockade activity at the *β*2AR, and carvedilol-stimulated *β*-arrestin–mediated *β*2AR signaling functions such as ERK phosphorylation and receptor trafficking. Notably, we also describe the identification of a cmpd-6 analog, A9, which displays a complete switch in the allosteric properties from a PAM to a classic NAM and yet retains the distinctive positive cooperativity exclusively with carvedilol at the *β*2AR.

Ligands that bias GPCRs toward preferentially activating either G-protein– or *β*-arrestin–mediated signaling hold immense therapeutic potential. Carvedilol is unique among *β*-blockers used in medicine in that it facilitates *β*-arrestin–biased signaling (unlike other *β*-blockers) while still blocking the deleterious effects of chronic Gs-mediated cAMP signaling downstream of activated *β*-adrenergic receptors (Reiter et al., 2012; Whalen et al., 2011). Indeed, findings from our competition radioligand binding as well as direct binding of ^3^H-carvedilol indicate that cmpd-6 further potentiates the cooperativity between carvedilol and *β*-arrestin1, but not that with Gs. Previous studies have shown carvedilol stimulation resulting in ERK activation downstream of *β*1 and *β*2ARs in a *β*-arrestin–dependent manner (Luttrell et al., 2018; Wang et al., 2017; Wisler et al., 2007). In the case of the *β*1AR this is through GPCR-mediated transactivation of the epidermal growth factor receptor (Kim et al., 2008; Noma et al., 2007). This signaling is implicated to be cardioprotective by counteracting G-protein–dependent catecholamine-induced toxicity and apoptotic pathways (Wang et al., 2018). Notably, clinical studies suggest that carvedilol may be superior to other *β*-blockers (such as metoprolol and propranolol) in preventing heart failure exacerbations and improving overall mortality in patients with reduced heart function. Carvedilol thus continues to be the drug of choice to treat patients with myocardial infarction and heart failure (Bristow et al., 1996; Colucci et al., 1996; Foody et al., 2002). The cardioprotective effects of carvedilol may be attributable to its unique ability to activate *β*-arrestin–mediated signaling pathways while potently blocking Gs activation (see accompanying manuscript by Wang et al., 2021). Interestingly, our findings show that cmpd-6 potentiates the ability of carvedilol, but not that of metoprolol, to block epinephrine-stimulated activation of Gs and cAMP generation. Additionally, cmpd-6 also augments the potency of carvedilol to stimulate *β*2AR-mediated ERK phosphorylation, which is known to be *β*-arrestin–dependent and involved in cytoprotective signaling. These findings highlight the allosteric potential of cmpd-6 in positively augmenting the desirable signaling properties of carvedilol. Although carvedilol represents a prototypic *β*-arrestin–biased orthosteric drug at the *β*-ARs, allosteric regulation of its varied signaling functions by cmpd-6 further expands the possibilities of developing improved *β*-blocker therapeutics even for biased orthosteric ligands. Indeed, by using a murine model of myocardial infarction, Wang et al. (2021) quite remarkably demonstrate the potential clinical implications of the unique positive cooperativity between cmpd-6 and carvedilol.

In addition to desensitizing G-protein–mediated signaling, *β*-arrestins are also known to play a pivotal role in receptor endocytosis. Over the past decade it has become evident that *β*-arrestins are recruited to membrane proteins (GPCRs, receptor tyrosine kinases (RTKs), and even ion channels) where they interact with or serve as scaffolds for components of the cellular endocytic machinery such as clathrin and adaptor protein 2 (AP2). *β*-arrestins thus function as key endocytic adaptors for activated GPCRs to facilitate their internalization resulting in either recycling or lysosomal degradation of the receptors (Freedman and Lefkowitz, 1996; Kovacs et al., 2009; Shenoy and Lefkowitz, 2011). Uniquely, carvedilol (in contrast to other *β*-blockers) displays pharmacological properties that are akin to *β*-agonists with respect to *β*-arrestin–mediated signaling. Carvedilol stimulation of cells results in G-protein–coupled receptor kinase 6–mediated phosphorylation of the *β*2AR as well as ubiquitination of the receptor by the E3 ligase, MARCH2 (membrane-associated RING-CH2) (Han et al., 2012). These signaling events have been reported to precede receptor internalization. In primary vascular smooth muscle cells, prolonged carvedilol treatment has been shown to trigger lysosomal trafficking and degradation of the *β*2AR. Consistent with these studies, we show that cmpd-6 potentiates carvedilol-stimulated loss of cell-surface *β*2AR leading to endocytosis and trafficking of the receptor to lysosomes. Although the effect of cmpd-6 on the above-noted post-translational modifications of the *β*2AR was not tested directly, our results on receptor endocytosis and lysosomal trafficking suggest that cmpd-6 enhances these carvedilol-stimulated responses.

Although cmpd-6 was identified to be a highly selective PAM for the *β*2AR (Ahn et al., 2018), our current findings clearly indicate that the cooperativity between cmpd-6 and carvedilol is also preserved at the *β*1AR. This property of cmpd-6 deviates from the usual pattern of receptor subtype selectivity that is a hallmark of allosteric modulators. Previous structural work from our group suggests that the binding site of cmpd-6 in the agonist-occupied *β*2AR is conserved in the *β*1AR, with different key residues mediating the receptor subtype-specific allosteric effect of cmpd-6 (Liu et al., 2019). In the absence of any structural data on the exact binding site of cmpd-6 to either the carvedilol-bound *β*1AR or *β*2AR, it may be speculated that the site could topologically overlap with the binding site of cmpd-6 as that in the agonist-bound *β*2AR. Although a given GPCR can have multiple, topologically distinct allosteric sites, it is also plausible that the binding site of cmpd-6 resides in a highly conserved structural motif that is common in this receptor family.

An emerging paradigm in GPCR structural biology is that GPCRs exist as ensembles of interconvertible inactive and active states, which are in conformational equilibrium (Hilger et al., 2018; Kobilka and Deupi, 2007; Manglik et al., 2015). Compared with canonical *β*-blockers, carvedilol has been shown to elicit unique conformational changes in the *β*2AR. In particular, quantitative proteomic studies (Kahsai et al., 2011) on labeling of solvent accessible reactive lysine and cysteine residues in the *β*2AR as well as findings from ^19^F-NMR studies on TM7 dynamics (Liu et al., 2012) suggest a distinct conformational signature of the *β*2AR when bound to carvedilol. These results also accord with the unique *β*-arrestin–biased agonism of carvedilol compared with other *β*-blockers (Kim et al., 2020; Wisler et al., 2007), which presumably is displayed only by a minor fraction of the receptor population within the conformational spectrum of carvedilol-bound receptor. Based on the data presented herein, cmpd-6 appears to stabilize a carvedilol-bound distinct conformational signature of the *β*2AR. Thus, cmpd-6 is a uniquely suited allosteric tool for examining the conformational dynamics of carvedilol-bound *β*2AR. 

Interestingly, our data show that cmpd-6 has absolutely no cooperativity with the antagonist/inverse agonist, carazolol. Although both ligands share an identical carbazole head-group, carvedilol differs from carazolol in having an extended aliphatic tail terminating with an anisole ring. From previously reported crystal structures of carvedilol- and carazolol-bound *β*2AR it is clear the carbazole moiety, which is shared by both ligands, occupies the orthosteric site situated deep in the transmembrane core (Bokoch et al., 2010; Cherezov et al., 2007; Ishchenko et al., 2019). However, a striking feature in the structure of carvedilol-bound *β*2AR is that the extended tail of carvedilol resides at a site distant from the deep orthosteric pocket. Based on this distinct binding modality of carvedilol, it may be hypothesized that the allosteric cooperativity between cmpd-6 and carvedilol is, in part, driven by the tail interactions of carvedilol with the *β*2AR. To discern the structural mechanism underlying this unique cooperativity, it will be of great interest to obtain atomic-level information on carvedilol-bound *β*2AR in complex with cmpd-6. Such structural studies will be useful not only for uncovering nuances of GPCR allostery but also for expanding our current understanding of the dynamic nature of GPCR allosteric sites and biased agonism.

The ideal allosteric drug would cooperatively interact with orthosteric ligands to selectively potentiate signaling pathways of therapeutic importance (Christopoulos, 2014; Thal et al., 2018). Although cmpd-6 does augment the putative cardioprotective effects of carvedilol mediated by *β*-arrestin signaling (shown in accompanying manuscript by Wang et al., 2021), it also potentiates agonist-stimulated G-protein signaling at the *β*2AR. This agonist-mediated signaling of cardiac *β*-receptors in part underlies the pathophysiology of heart failure. As such, from a potential therapeutic perspective, the dual cooperativity of cmpd-6 with *β*-agonists and carvedilol at the *β*2AR is diametrically opposed. Previous structure-activity relationship studies with cmpd-6 led to the synthesis of several chemically modified analogs of the parent compound (Liu et al., 2019). In the current study, we show that the analog A9 has no cooperativity with agonists but still retains the unique positive allosteric cooperativity exclusively with carvedilol. A9 thereby serves as a small molecule prototype, which displays pharmacologic properties desirable of a potential allosteric therapeutic that together with carvedilol could be advantageous to abate cardiovascular ailments including heart failure.

In summary, our study describes the unique ability of cmpd-6 and its analog A9 to allosterically potentiate the pharmacologic properties of carvedilol, a key cardiovascular therapeutic. The discovery of this unexpected interaction has direct therapeutic implications and also serves to advance our understanding of GPCR allostery and biased agonism.

## Abbreviations

*β*-AR: *β*-adrenergic receptor
*β*arr1: *β*-arrestin1
*β*1AR: *β*1-adrenergic receptor
*β*2AR: *β*2-adrenergic receptor
BSA: bovine serum albumin
CI: confidence interval
cmpd-6: compound-6
DDM: n-dodecyl-*β*-d-maltopyranoside
DMEM: Dulbecco’s modified Eagle’s medium
ERK: extracellular signal-regulated kinase
GPCR: G-protein–coupled receptor
GsHet: Gs-*αβγ* heterotrimer
^3^H-Carv: [^3^H]-R/S-carvedilol
HBSS: Hanks’ balanced salt solution
HDL: high-density lipoprotein
^125^I-CYP: [^125^I]-cyanopindolol
MEM: minimum Eagle’s medium
NAM: negative allosteric modulator
Nb6B9: nanobody-6B9
Nb80: nanobody-80
PAM: positive allosteric modulator
^35^S-GTP*γ*S: guanosine 5*γ*-*O*-(3-[^35^S]thio)triphosphate
SEC: size-exclusion chromatography
SPA: scintillation proximity assay
V2R: vasopressin 2 receptor
YFP: yellow fluorescent protein
YSi: yttrium silicate
